# A systematic review of longitudinal studies on the association between depression and smoking in adolescents

**DOI:** 10.1186/1471-2458-9-356

**Published:** 2009-09-22

**Authors:** Michael O Chaiton, Joanna E Cohen, Jennifer O'Loughlin, Jurgen Rehm

**Affiliations:** 1 Dalla Lana School of Public Health, University of Toronto, Toronto, Canada; 2 Centre de Recherche du Centre hospitalier de l'Université de Montréal, Department of Social and Preventive Medicine, Université de Montréal, Montreal, Canada; 3 Ontario Tobacco Research Unit, University of Toronto, Toronto, Canada; 4 Addiction Research Institute, Zürich, Switzerland; 5 Centre for Addiction and Mental Health, Toronto, Canada

## Abstract

**Background:**

It is well-established that smoking and depression are associated in adolescents, but the temporal ordering of the association is subject to debate.

**Methods:**

Longitudinal studies in English language which reported the onset of smoking on depression in non clinical populations (age 13-19) published between January 1990 and July 2008 were selected from PubMed, OVID, and PsychInfo databases. Study characteristics were extracted. Meta-analytic pooling procedures with random effects were used.

**Results:**

Fifteen studies were retained for analysis. The pooled estimate for smoking predicting depression in 6 studies was 1.73 (95% CI: 1.32, 2.40; p < 0.001). The pooled estimate for depression predicting smoking in 12 studies was 1.41 (95% CI: 1.21, 1.63; p < 0.001). Studies that used clinical measures of depression were more likely to report a bidirectional effect, with a stronger effect of depression predicting smoking.

**Conclusion:**

Evidence from longitudinal studies suggests that the association between smoking and depression is bidirectional. To better estimate these effects, future research should consider the potential utility of: (a) shorter intervals between surveys with longer follow-up time, (b) more accurate measurement of depression, and (c) adequate control of confounding.

## Background

Co-occurrence of tobacco use and depression has long been a major concern because of the substantially increased risk to health.[1] Most [2-12] but not all [13-15], cross-sectional studies report a strong positive association between smoking and depression. Furthermore, how these health risks inter-relate temporally remains a topic of debate. Specifically, there is no consensus on whether smoking causes depression or depression causes smoking in the natural course of tobacco use and depression.[16]

Several hypotheses have been put forward to explain the possible mechanisms underlying the association between smoking and depression. The "self-medication" hypothesis suggests that depression may lead to smoking because people smoke to relieve symptoms of depression. [17-20] In contrast, other authors have suggested that cigarette smoking causes depression as nicotine exposure may damage neurochemical pathways, such as monoamine neurotransmission. [21-23] Bi-directional mechanisms in which smoking and depression reinforce each other have also been postulated.[12,23,24] Finally, Kendler (1993) hypothesizes that there is no causal relationship and that the perceived association is a function of underlying shared environmental and genetic risk factors.[24]

Adolescence is regarded as the key period for the study of the onset of smoking and depression co-occurrence. The period of adolescence represents the peak of the incidence of both smoking and depression.[25] More specifically depressive symptoms tend to increase in early adolescence and then become stable or decline in later adolescence.[26] Similarly, most people who start smoking do so before the age of 18. To study the co-occurrence of smoking and depression it is necessary to investigate the association prior to onset.

While cross-sectional studies find that depression and smoking are associated among adolescents, longitudinal studies are needed to address temporality. Since Kandel and Davis [27] reported that depression in adolescents could lead to the onset of smoking, several longitudinal studies have assessed the relationship between smoking and depression in adolescents.[12,16,28] In previous reviews, Paperwalla et al. [12] and Schepis and Rao [28] examined the etiology of depression and smoking in general without conducting systematic search strategies or analysis. Park and Romer [16] conducted a systematic, descriptive analysis of cross-sectional and longitudinal studies published up to 2005. However, their review was limited by the lack of a quantitative component, and inclusion of several papers using data from the same cohort as independent studies.

This review focuses on longitudinal studies wherein temporality can be ascertained, and it provides a quantitative summary of estimates of the association between smoking and depression among adolescents.

## Methods

English language studies published between January 1990 and December 2007 were extracted from the PubMed, OVID, and PsychInfo databases using the following abstract keywords: (a) smoking, tobacco, cigarettes; (b) depress* (c) child*, adolescen*, youth; (d) longitudinal, cohort, follow-up. Titles and abstracts were then screened to determine if publications met the following inclusion criteria: (1) the association between smoking and depression was studied longitudinally; (2) the outcome (either the onset of depression or the onset of smoking) was measured when subjects were aged 13 -19 years; (3) the study population was broadly representative of adolescents (studies of clinic populations, the unemployed, the incarcerated, and pregnant girls were excluded); and (4) the effect of smoking on the onset of depression, or the effect of depression on the onset of smoking was reported explicitly as a risk, hazard, or odds ratio. Studies that examined related but distinct mental health constructs including anxiety, stress, and negative affect were not included. Additional studies that met the inclusion criteria were identified by scanning references in the reports retained in the primary search, and through searching the Internet through Google Scholar. For cohorts with multiple relevant publications, the latest publication with the longest follow-up that reported on outcomes in adolescents was selected to avoid duplication.

The following data were abstracted from each study retained: country in which the study had been conducted; sample size; year(s) of data collection; time period over which the relationship was estimated; participants' age/grade, the definition of smoking; the definition of depression; covariates included in the multivariate modeling; the analytic method; and the parameter estimate of the unadjusted association between smoking and depression (with 95% confidence intervals if available). Finally, the estimate of effect(s) after controlling for covariates was recorded. The quality of the studies included in this review was characterized across factors that may affect generalizability and internal validity in cohort studies.[29] Several studies reported only one direction of effect (i.e., smoking initiation was the only outcome tested). Studies that reported results only by subgroup (i.e., males and females) were included as separate studies in the pooled analysis.

### Synthesis

While there was considerable variability in methods of analysis and the description of results, we undertook a quantitative synthesis of data across studies. The log scale estimate and its standard error were entered into Stata 10.0 [30] and pooled using the 'meta' procedure with the random effects option. This procedure uses inverse variance weighting to combine the effect measures. The results for depression predicting the onset of smoking, and smoking predicting the onset of depression were tabulated separately, and forest plots were created for each analytic group.

### Sources of longitudinal studies

**Figure 1:**
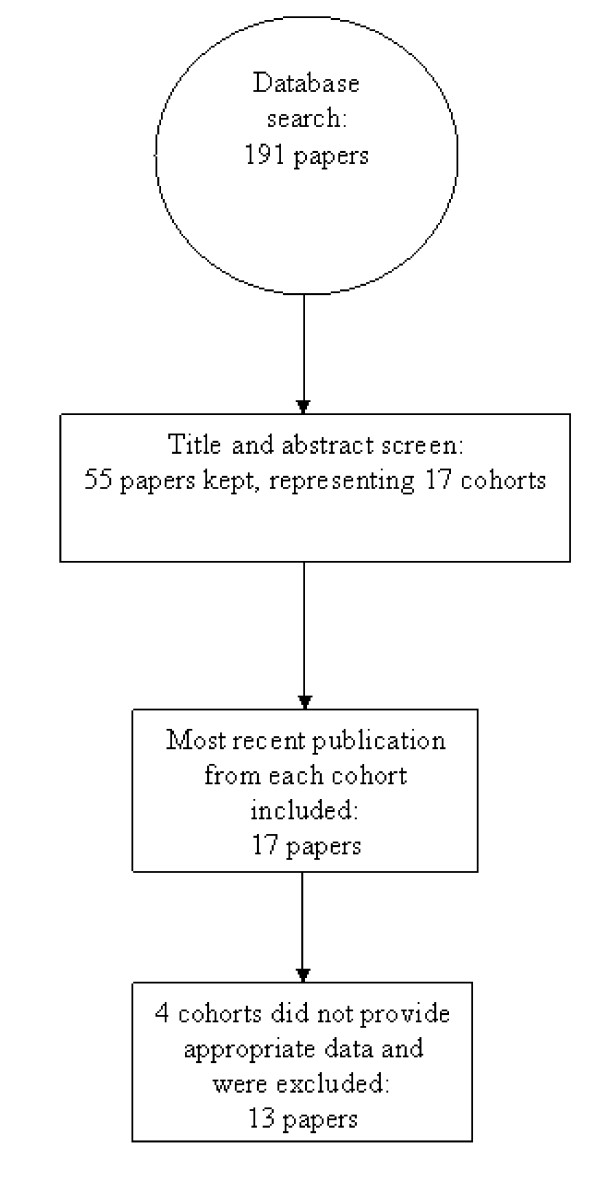
**Search strategy diagram**.

The database search yielded 191 publications. Eight additional reports were obtained through a search of references. Sixteen publications were excluded because they did not analyze the data longitudinally (i.e., they pooled data across data collection waves and analyzed the data as if they were cross-sectional). Screening of titles and abstracts for inclusion and exclusion criteria produced a total of 55 relevant publications representing 17 different cohorts. Four studies from separate cohorts [31-34] did not have data that could be extracted and were therefore excluded. Thirteen studies met the inclusion criteria (Figure 1).

### Study characteristics

Table 1 presents selected characteristics of the 13 studies retained. Study participants were drawn primarily from school-based samples except for the national longitudinal study of adolescent health[35] and the Teenage Attitudes and Practices Study (TAPs)[36] which were nationally representative population samples in the United States, and the Christchurch Health and development study which is a birth cohort.[37] Studies were conducted in the following geographic locations: the United States, Mid-Atlantic states, Michigan, Oregon, Northern Virginia, Los Angeles County, Albany, Pittsburgh, Australia, New Zealand, and Hong Kong. A variety of analytic approaches were used (i.e., logistic, ordinal logit, and Cox regression) which yielded differing types of estimates of effect. All studies were conducted as panel studies with interval censoring. Six studies had only one follow-up survey. Two studies collected follow-up data at 6-month intervals, six studies collected data at annual intervals, and five studies collected data at longer than one-year intervals. Five publications followed their cohorts in total for one year, while 5 followed for more than 3 years.

**Table 1:** Characteristics of longitudinal studies on the relationship between depression and smoking among adolescents.

**First Author**	**Year**	**Where study took place**	**Baseline age or grade**	**Follow-up period**	**n**	**Method of analysis**	**Estimate of effect of smoking predicting depression**	**Estimate of effect of depression predicting smoking**
Brown	1996	Western Oregon	14 to 18 years	1 year	1507	Logistic regression	OR = 1.89 (1.04, 3.45)	OR = 2.04 (1.16,3.60)
Escobedo	1998	United States	12 to 18 years	1 year	7885	Logistic	Not assessed	OR = 1.30 (1.10, 1.60)
Patton	1998	Victoria, Australia	Grades 9 to 12	every 6 months for 3 years	1688	person years/time varying Survival analysis	Not assessed	HR = 2.00 (1.30, 2.90) Significant among girls only
Wu	1999	Mid Atlantic US	8 and 9 years	Every 6 months, 1 year	1731	Survival analysis	HR = 1.66 1.28, 2.16	HR = 1.06 (0.74, 1.51)
Goodman	2000	United States	Grade 7 to 12	2 years	8704	Logistic	OR = 3.90 (1.85, 8.20)	OR = 1.72 (0.78, 3.81)
Albers	2002	Massachussets	12-15 years old	4 years	522	Logistic	OR = 1.74 (0.97, 3.14)	Not assessed
Fergusson	2003	New Zealand	Birth	every year, 21 years	1265	Incidence ratio	Not assessed	RR 1.75 (1.13, 2.70)
Lam	2004	Hong Kong	Grade 7	1 year	1894	Logistic	OR = 2.17 (1.40,3.36)	OR 1.48 (1.07, 2.05)
Audrain-McGovern	2004	Northern Virginia	Grade 9	Every year, 3 years	615	Ordinal logit	Not assessed	OR = 1.04 (0.76, 1.42)
Clark	2004	Pittsburgh	10 to 12 years	2 years, 3 years, 3 years	572	Survival analysis	Not assessed	HR = 1.0
Killen	1997	Northern California	Grade 9	1 year for 4 years	1901	Survival analysis	Not assessed	Girls: HR = 1.55 (n = 463 x2 = 4.91), boys: HR = 1.03 (n = 481, x2 = 7.84)
Gilpin	2004	California	12 to 15 years old	1 year	1764	Logistic	Not assessed	OR = 1.46 (1.04, 2.05)
Brook	1998	Albany and Saratoga	1 to 10 years	3 years, 6 years, 5 years	975	Logistic	OR = 1.19 (1.01, 1.40)	OR = 1.12 (0.28, 4.44)

OR = odds ratio

HR = hazard ratio

RR = risk ratio

### Internal validity and generalizability

Studies retained for analysis varied on a number of criteria measuring "quality" that may have affected internal validity or generalizability (See additional file 1: qualitytable.xls for more details). Most studies used populations randomly selected from the population or a school sampling frames--except Clark and Cornelius [38] who recruited a convenience sample. Albers et al. [11] and Gilpin et al. [39] had loss to follow up proportions greater than 25%. All studies controlled at least for age and sex; the range of other variables controlled for in multivariate analysis varied considerably across studies (Table 2).

**Table 2:** Covariates included in final multivariate models reported in 13 longitudinal studies that examined the association between smoking and depression in adolescents.

**Covariate**	**Study(ies) that included the covariate in the final multivariate model (citation number)**
Age	(all)
Sex	(all)
alcohol use	[26,32,33,37,45,46,65]
parental education	[32,36,41,42,61,66]
family income	[32,37,41,61,66]
race/ethnic group	[32,35-37,42,46,65]
smoking among peers	[26,32,33,37,45,65,66]
parental smoking	[26,32,36,66]
marijuana/other drug use	[32,33,41,65]
Academic performance	[32,36,37,65]
physical activity	[26,65]
temperament	[33]
social support	[33]
parental attachment	[37]
Anxiety	[31,37]
Childhood adversity	[37]
conduct problems	[37,66]
novelty-seeking	[37]
neuroticism	[37]
drive for thinness	[45]

Table 3 describes indicators used to measure smoking in the 13 studies retained for analysis. Smoking onset was typically described as one puff on a cigarette. The definition of a "smoker" ranged from "had smoked a single cigarette" to "daily smoking". Some studies defined a smoker using progression in stage of smoking including smoking a first cigarette or an experimenter becoming a daily smoker. Two studies included measures of smoking intensity such as number of cigarettes per day.[26,37] Of the six studies that examined onset of depression, three started from a baseline population of never smokers, while the others used baseline smoking to predict depression. Because the number of studies available for comparison was limited, we could not determine if or how the measure of smoking used affected the estimate of the association with depression.

**Table 3:** Measures of smoking and depression used in 13 longitudinal studies that examined the association between smoking and depression in adolescents

**Measures of smoking**	**Study(ies) that used the measure**
One puff	[11,39,45,65]
One cigarette	[26,27,34,38,46,48,76]
Ever (over 100 cigarettes)	[36]
No cigarettes in past 6 months	[33]
Last 30 days	[35,48,72]
Daily smoking	[26]
Total months of use	[27]
No. cigarettes per day	[37]
Three or more cigarettes per week	[42]
DSM nicotine dependence	[37]
	
**Measures of depression**	**Study(ies) that used the measure**

**Structured or semi structured**	
Kiddie-Schedule for Affective Disorders	[42]
DIS for Children	[41]
DSM-III-R	[38]
Child and Adolescent Psychiatric Assessment	[31,66]
High Clinical Interview Schedule	[26]
**Symptomatological**	
CES-D	[33,35,45,65]
Depressed mood questions (unknown scales)	[32,39,46]
Mellinger Scale	[34,36,71]

Major Depressive Disorder is defined in the *Diagnostic and Statistical Manual of Mental Disorders *[40] as a period of at least two weeks characterized by at least five severe and persistent depressive symptoms. The symptoms must not represent a normal reaction to the death of a loved one and should not be due to an identifiable organic factor such as a physical illness or drug exposure. While clinical exams and structured interviews are the gold standards of measurement[12], they tend to be too lengthy and complex for use in large-scale longitudinal studies. Despite measurement challenges, five studies used semi-structured interview based measures of depression.[26,38,41,42] The majority of studies however used measures of depressive symptomatology (Table 3), the most common of which were the Center for Epidemiology Depression Scale (CES-D) and the Mellinger Scale.[27,43-45] Three studies examined past history of mental illness or depression.

## Results

### Descriptive Analysis

Six studies investigated smoking and depression as both outcomes and predictors, allowing for direct assessment of the bidirectionality of the association in the same study population. Two of these six studies [41,46] reported that smoking was a significant predictor of depression, but depression did not predict smoking. All six bidirectional studies found that smoking was predictive of depression; however, two studies concluded that depression predicting smoking was a better fit of the data.[26,37] While Fergusson et al. did not present risk estimates for smoking predicting depression, the authors concluded that the data best fit the model that depression led to smoking.[37] Brown (1996) and Lam (2004) in longitudinal surveys of youth in Oregon and Hong Kong, respectively, all concluded that depression predicted smoking or progression of smoking, while increased smoking predicted increased depression.[33,42,46-48]

Of the six studies that examined the bidirectionality of the association between smoking and depression, the three that used a clinical measure of depression [37,41,46] found stronger effects for depression predicting smoking than for smoking predicting depression. In the other three studies that used a symptomatological measure of depression, the reverse was true.[35,46,48] Self-report measures of depressive symptomology tend to overestimate prevalence compared to semi-structured clinical interviews.[28]

Several studies indicate that there may be effect modification in the association and have identified different subgroups in which the relationships between smoking and depression differ. Three studies found that the association of smoking and depression was only significant among females.[26,34,45] Patton (1998) found that depression was predictive of smoking only in youth who reported that all or most of their friends smoked.[26]

### Pooled analysis

**Figure 2:**
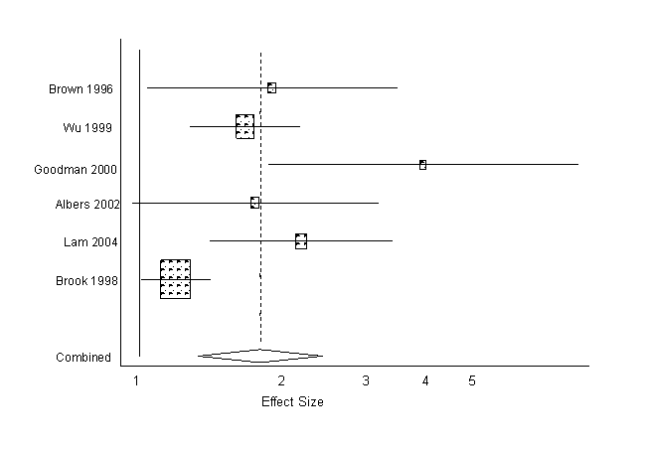
**Effect sizes and 95% confidence intervals reported in six longitudinal studies on smoking predicting depression in adolescents**.

**Figure 3:**
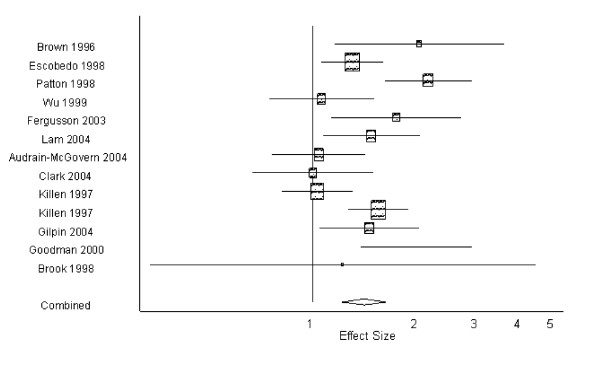
**Effect sizes and 95% confidence intervals reported in 12 longitudinal studies on depression predicting smoking in adolescents**.

The pooled estimate for smoking predicting depression in the six studies with available data was 1.73 (95% CI: 1.32, 2.40; p < 0.001) (Figure 2). The pooled estimate for the 12 studies that examined depression predicting smoking was 1.41 (95% CI: 1.21, 1.63; p > 0.001) (Figure 3). There was no significant publication bias according to either Begg's Test or Egger's Test for both depression and smoking outcomes. Visual inspection of the bias plot (not shown) was consistent with this statistical finding. There was statistical heterogeneity in studies predicting depression and those predicting smoking according to the Q test for heterogeneity (p = 0.08), and therefore a random effects model was used for the meta-analysis of the data. Removal of the largest study [36] predicting smoking did not dramatically affect the estimate (1.37 (95% CI: 1.17, 1.60)).

## Discussion

The present review suggests that there is evidence in longitudinal studies of adolescents that depression predicts smoking, and that smoking predicts depression. The pooled estimates for depression predicting smoking (1.41 (95% CI: 1.21, 1.63)) and smoking predicting depression (1.73 (95% CI: 1.32, 2.40)) were both statistically significant despite the relatively small number of studies and wide variability in study designs and measures.

This analysis revealed moderately strong evidence supporting a relationship whereby smoking predicts depression among adolescents. Only one study [11] found that smoking did not significantly predict depression, but this study had the smallest sample size of the studies included, and the magnitude of the effect was large (1.74). The magnitude of the pooled effect for smoking predicting depression was also larger than that of depression predicting smoking. There is evidence that smoking leads to dysregulation of the hypothalamic-pituitary-adrenal system and hypersecretion of cortisol [49], which can regulate biological and psychological reactions to sources of stress. [50-52] Consequently, smoking might interfere with natural adaptive coping mechanisms and induce depression.[53]

Conversely, the results also may be interpreted to suggest that depression leading to smoking is the more likely pathway. Smokers may use nicotine as a means of 'self-medicating' symptoms such as depressed mood and fatigue that are related to depression; nicotine has been shown to produce an elevation in mood.[24] Nicotine may also have anti-depressant properties, acting in a similar manner to common antidepressants through the serotonin neurotransmitters. [54-56] In the few studies that tested competing models directly, depression predicting smoking was the stronger effect. When more specific measures of depression are used, depression is better able to predict smoking, suggesting that random misclassification of depression may bias the effect of depression predicting smoking towards the null in some studies. Furthermore, smoking may be more easily quantified and recalled by adolescents--more studies measure previous smoking history (i.e., identify never smokers) and control for it than measure previous depression or control for previous depressive episodes. If depression at a young age went undiagnosed at the time, it is possible and perhaps likely that previous depression would have been forgotten or mischaracterized. While it is possible to forget smoking experiences, smoking is a tangible event compared to a depressive episode. Controlling for previous smoking without controlling for previous depression will create bias by design.

The current analysis cannot refute the hypothesis that the relationship between smoking and depression is spurious. It is possible that if the two conditions are causally related, the relationship may reflect several potential causal mechanisms in which: (a) depression precedes smoking, (b) smoking precedes depression, (c) there is a bidirectional relationship between smoking and depression, (d) the relationship is a function of unknown risk factors, or (e) there are subgroups in which the relationship between smoking and depression differs. To address these issues, the nature of the relationship between depression and smoking should be investigated further. While temporality is an important condition for causality, there are other issues in the current literature of prospective studies that limit our ability to draw robust conclusions about the nature, causal or otherwise, of the relationship between smoking and depression. These issues should inform future research and are discussed below.

### Misclassification

The conceptualization, the definition and the method of measurement of exposure can affect the interpretation of an association. Studies that used a clinically based definition of depression were generally consistent in showing that the magnitude of effect was greater for depression predicting smoking than smoking predicting depression; however, only 5 studies used diagnostic interviews to measure depression. Measures of depressive symptomatology may have more random measurement error than diagnostic interviews, attenuating parameter estimates towards the null. Furthermore, cut-points for depressive symptomatology scales unnecessarily discard information.

Interval censoring may affect the interpretation of the temporal sequence of events if both depression and smoking occur during the same interval. For example, smokers may experience rapid fluctuations in mood (compared to non-smokers), as result of intermittent periods of withdrawal, followed by short-term relief after smoking.[57] These rapid fluctuations may cause misclassification of the magnitude of depression symptoms depending on the precise timing of administration of the survey. Shortening the length of the interval will ameliorate this issue.

### Selection bias

Sample selection may introduce bias. A previous history of depression increases the risk of future depression, which suggests that estimates should be adjusted for a history of depression.[25] Selecting adolescents without smoking or depression at study entry reduces the need to control for the possible effects of a history of either depression or smoking. However, selecting such a subgroup may also introduce bias by studying a unique subgroup whose initiation of smoking or depressive symptoms is potentially unusually delayed compared to a 'normal' population. This subgroup perhaps represents a distinct population where the relationship between smoking and depression differs from a representative population.

### Unmeasured confounding

The magnitude of the association between depression and smoking has been shown to be substantially reduced after controlling for such variables as school performance, socio-economic status, and alcohol use.[42,58-60] Duncan et al. argued that these variables (conceptualized in these analyses as confounders) might in fact be indicators of depression; and therefore it is inappropriate to include them in the model.[61] Nevertheless, this current review shows that these variables are unlikely to be mediators as they do not consistently or entirely attenuate the relationship; however, many studies did not control for the entire range of covariates suggested as possible confounders. It is also conceivable that other unknown or unmeasured common factors are responsible for a spurious relationship between depression and smoking. The identification of whether potential covariates are confounders of the depression and smoking relationship or mediators is a key question for future research. Techniques such as pathway analysis or structured equation modeling could be used to test for mediation.[62] Direct acyclic graphing may also be useful to help select appropriate confounders.[63,64]

### Subgroups with different causal pathways

The evidence is consistent with a hypothesis that the association between smoking and depression may differ among different subgroups. There is some indication that the relationship between depression and smoking is specific to females or among youth with friends who smoke.[26,34,45] There may also be undefined subgroups in which smoking leads to depression while in others, depression leads to smoking--an hypothesis that is consistent with the results of the present study. Audrain McGovern et al. report that there was a significant relationship of smoking and depression only among adolescents with variants of DRD2, a dopamine receptor gene.[65] In a twin study, Silberg found that genetic influences were only pronounced among females for progression to smoking, and that for males, environmental influences, particularly family and peer smoking, explained the variance between smoking and depression.[66] As well, the association of depression and smoking may vary based on contextual factors such as prevalence of depression or smoking in the population and social acceptability of smoking.[67,68] The inconsistency across studies examining smoking and depression may relate to differences in environmental and subgroup variations.

## Limitations

This paper used strict inclusion criteria to include as homogenous a population of studies as possible in order to perform the pooled analysis. As such, studies that used techniques such as growth curve modeling [33,69,70], cross-lagged analysis [71], or correlations[34,72] were not included. While broader would have included more studies and different perspectives, the advantage of a focused research question allows the important information to be highlighted and quantified in a way that a broader survey could not.

The pooled estimates should be interpreted with caution because they are based on studies with heterogeneous measures of exposure and outcomes, and differing study designs. Estimates adjusted for covariates were selected from each study, but the variables included to control for confounding varied from study to study. There may therefore be residual confounding of the pooled estimate, which could inflate the estimate away from the 'true' value.[73]

Several studies were excluded because the outcome was measured in young adulthood rather than adolescence.[47,74] It could be argued that the young adult measure is more acceptable as it reduces the number of censored observations. However, one major purpose of studying adolescents is to capture the initiation of the relationship between smoking and depression. The strength of the association between smoking and depression is well established before age 20 and consequently the initiation of the relationship must be established by this point. Dramatic developmental and neurobiological changes in the adolescent brain may influence drug use vulnerability and future susceptibility to depression and, consequently, the likelihood that an association exists may differ across ages.[75]

## Conclusion

While the association between smoking and depression is not in doubt, the reasons underlying the association matter both for those affected by both depression and smoking, but also for the development and implementation of interventions. Understanding the causal mechanisms underlying the relationship could allow for the development of interventions for adolescents with these conditions, and possibly prevent the implementation of interventions based on an erroneous causal mechanism that may have negligible or even iatrogenic effects. If, for example, some individuals smoke in order to control symptoms of depression, interventions that focus solely on smoking could be ineffective, or indeed, potentially dangerous. However, if smoking exacerbates depression symptoms, excluding those who are depressed from treatment could lead to further harm. Further research into identifying the nature of the association should be initiated.

## Competing interests

The authors declare that they have no competing interests.

## Authors' contributions

MC performed the literature search, the quantitative analysis and drafted the manuscript. JEC, JOL, JR supervised the search and contributed to the conceptualization of the project including statistical design. All authors read and approved the final manuscript.

## Pre-publication history

The pre-publication history for this paper can be accessed here:



## Supplementary Material

Additional file 1 **Quality table**. Quality Assessment of included longitudinal studies on the association of depression and smoking. Click here for file
